# Ultrasonographic Assessment of Hepatic Capsular Thickness in Fitz–Hugh–Curtis Syndrome: Correlation with Computed Tomography

**DOI:** 10.3390/tomography12060079

**Published:** 2026-05-27

**Authors:** Ye Jun Park, Eun Ju Yoon, Jun Hyung Hong, Eai Hong Hwang, Tae-Hoon Kim, Seong-Jung Kim, Soo-Min Heo, Hyun Chul Kim, Sang Gook Song, Jin Woong Kim

**Affiliations:** 1 Department of Radiology, Chosun University Hospital and Chosun University College of Medicine, Gwangju 61452, Republic of Korea; yejun0524@gmail.com (Y.J.P.); racidian@gmail.com (E.J.Y.); khcikim@chosun.ac.kr (H.C.K.); sgsong71@gmail.com (S.G.S.); jw4249@gmail.com (J.W.K.); 2 Medical Research Institute, Chosun University, Gwangju 61452, Republic of Korea; ygegh@hanmail.net (S.-J.K.); he0s00min@chosun.ac.kr (S.-M.H.); 3 Department of Radiology, Miraero 21 Medical Center, Gwangju 62045, Republic of Korea; mplife@daum.net; 4 Department of Radiology, Chosun University Hospital, Gwangju 61452, Republic of Korea; tae_hoonkim@hanmail.net; 5 Department of Internal Medicine, Chosun University Hospital and Chosun University College of Medicine, Gwangju 61452, Republic of Korea; 6 Department of Obstetrics and Gynecology, Chosun University Hospital and Chosun University College of Medicine, Gwangju 61452, Republic of Korea

**Keywords:** Fitz–Hugh–Curtis syndrome, pelvic inflammatory disease, hepatic capsular thickness, computed tomography, ultrasonography

## Abstract

Objectives: To investigate whether hepatic capsular thickness (HCT) measured on ultrasonography (US) is associated with HCT measured on arterial-phase computed tomography (CT), and to evaluate the potential discriminative performance of US-measured HCT in women with Fitz–Hugh–Curtis syndrome (FHCS). Methods: In this retrospective dual-center case–control study, 17 women with clinically diagnosed FHCS who underwent both arterial-phase CT and abdominal US within a 3-day interval were included. Thirty-five healthy women served as controls. HCT was measured on CT and US by two abdominal radiologists blinded to clinical information. HCT values were compared between groups, the association between CT and US measurements was assessed, interobserver agreement was evaluated using the intraclass correlation coefficient (ICC), and receiver operating characteristic analysis was performed to explore candidate cutoff values for discriminating FHCS from controls. Results: Median HCT on CT was significantly greater in the FHCS group than in the control group [1.80 mm (IQR, 1.60–2.00) vs. 0.60 mm (IQR, 0.40–0.70); *U* = 595.0, *p* < 0.001]. Median HCT on US was also significantly greater in the FHCS group than in the control group [1.50 mm (IQR, 1.30–2.00) vs. 0.70 mm (IQR, 0.60–0.80); *U* = 589.0, *p* < 0.001]. CT- and US-based HCT measurements showed a significant positive correlation (rho = 0.66, *p* < 0.001). Interobserver agreement for HCT measurement was good in the overall cohort (ICC, 0.804; 95% confidence interval [CI], 0.66–0.89). In exploratory receiver operating characteristic (ROC) analysis, the candidate cutoff values were 1.1 mm for CT and 0.85 mm for US. These ROC-derived metrics should be interpreted as exploratory estimates from an idealized case–control setting rather than as real-world diagnostic performance. Conclusions: US-measured HCT was significantly increased in women with clinically diagnosed FHCS and showed a significant positive correlation of moderate strength with CT-measured HCT. These findings suggest that US-based HCT assessment may provide supportive imaging information in patients with suspected FHCS. Further validation in larger cohorts, particularly in clinically relevant control populations, is warranted.

## Simple Summary

Fitz–Hugh–Curtis syndrome is an inflammation of the liver lining often associated with pelvic infections in women, causing severe upper abdominal pain. Currently, CT scans are used for diagnosis, but they involve radiation and contrast agents. This study suggests that measuring the thickness of the liver’s outer layer using ultrasound may help identify this condition. The findings indicate that these ultrasound measurements might correlate with results from CT scans. These findings suggest that ultrasound-based measurement of hepatic capsular thickness may provide supportive imaging information in patients with suspected Fitz–Hugh–Curtis syndrome and warrant further validation in larger, clinically relevant populations.

## 1. Introduction

Fitz–Hugh–Curtis syndrome (FHCS) is a form of perihepatitis associated with pelvic inflammatory disease (PID), most commonly related to Chlamydia trachomatis or Neisseria gonorrhoeae infection [1,2,3]. Clinically, patients often present with right upper quadrant pain, with or without accompanying pelvic pain, fever, or vaginal discharge [4,5,6,7,8,9]. Because these symptoms may overlap with those of other hepatobiliary or peritoneal conditions, establishing the diagnosis can be challenging in routine practice.

Computed tomography (CT), particularly arterial-phase imaging, has been reported to be useful in the evaluation of FHCS, with hepatic capsular enhancement along the anterior liver surface being a well-described imaging finding [1,2,4]. However, CT has several practical limitations, including ionizing radiation exposure, contrast material administration, cost, and limited availability in some clinical settings.

Ultrasonography (US) is widely available, radiation-free, and commonly used as a first-line imaging modality in young women presenting with abdominal pain. Nevertheless, the ultrasonographic findings of FHCS have not been well standardized. Previous reports have primarily described ancillary findings, such as perihepatic or pelvic fluid collection, violin-string adhesions, or nonspecific perihepatic soft tissue thickening, mostly in isolated cases or small series [5,10,11,12,13,14]. Quantitative US criteria that can be compared with CT-based findings have not been sufficiently explored.

In this context, hepatic capsular thickness (HCT) may represent a measurable imaging marker of perihepatic inflammation. Therefore, the purpose of this study was to compare HCT measured on US and arterial-phase CT in women with clinically diagnosed FHCS and to explore whether US-measured HCT may provide supportive imaging information in patients with suspected FHCS.

## 2. Materials and Methods

### 2.1. Study Design and Patients

This retrospective dual-center case–control study was approved by the institutional review boards of the participating institutions (CHOSUN 2025-08-013-001), and the requirement for informed consent was waived because of the retrospective study design.

We reviewed the medical records of 1237 patients who were clinically suspected of having PID over a 10-year period. Among them, 17 women (median age, 20.0 years; IQR, 22.0–32.0 years) with clinically diagnosed FHCS were included. FHCS was diagnosed clinically and radiologically on the basis of compatible acute abdominal pain, including typical right upper quadrant pleuritic pain, leukocytosis, clinical improvement after antibiotic treatment, and characteristic hepatic capsular enhancement on arterial-phase CT [4,5,8]. Eligible patients were required to have undergone both arterial-phase CT and abdominal US within a 3-day interval; those with missing data or an interval exceeding 3 days were excluded. Immunoserologic testing was performed in only one patient, and cervical culture or polymerase chain reaction testing was not available in this cohort. Therefore, FHCS was defined clinically and radiologically rather than on the basis of uniform microbiologic confirmation.

The control group consisted of 35 healthy, sexually active women (median age, 40.0 years; IQR, 35.5–45.0 years) selected from an institutional database. These individuals had undergone the same imaging protocol for routine health screening or preoperative kidney donor evaluation. Because this was a retrospective study requiring both arterial-phase CT and abdominal US within a short interval, healthy controls were used as the comparison group.

### 2.2. Imaging Acquisition

CT examinations were performed using multidetector CT systems from multiple vendors, including 64-channel MDCT (LightSpeed VCT, GE Healthcare, Milwaukee, WI, USA), Helical CT (GE HiSpeed Advantage, GE Healthcare, Milwaukee, WI, USA), 16 MDCT (Somatom Sensation, Siemens Healthineers, Erlangen, Germany), and 640 MSCT (Aquilion ONE, Toshiba Medical Systems, Otawara, Japan). After intravenous administration of 100–150 mL of contrast material (Ultravist 370, Bayer AG, Leverkusen, Germany) at a rate of 3.5 mL/s, arterial-phase images were obtained with a slice thickness of 3–5 mm.

US examinations were performed using high-resolution systems (LOGIQ 7 and 9, GE Healthcare, Milwaukee, WI, USA; Sequoia 512, Acuson, Mountain View, CA, USA; iU22, Philips Healthcare, Bothell, WA, USA) equipped with broad-bandwidth curved and linear transducers. Curved transducers were used for general abdominal scanning, whereas linear transducers were additionally used when the anterior hepatic capsular complex could be more clearly delineated at a shallow depth. When available, the transducer that provided the clearest depiction of the anterior hepatic capsular complex was used for measurement. Intercostal scanning was used to visualize the anterior hepatic surface.

### 2.3. Image Analysis

Two abdominal radiologists, blinded to clinical information, independently measured anterior hepatic capsular thickness on magnified arterial-phase CT images and corresponding US images displayed on the picture archiving and communication system (Infinitt PACS, version 7.0; Infinitt Healthcare, Seoul, South Korea).

**Figure 1:**
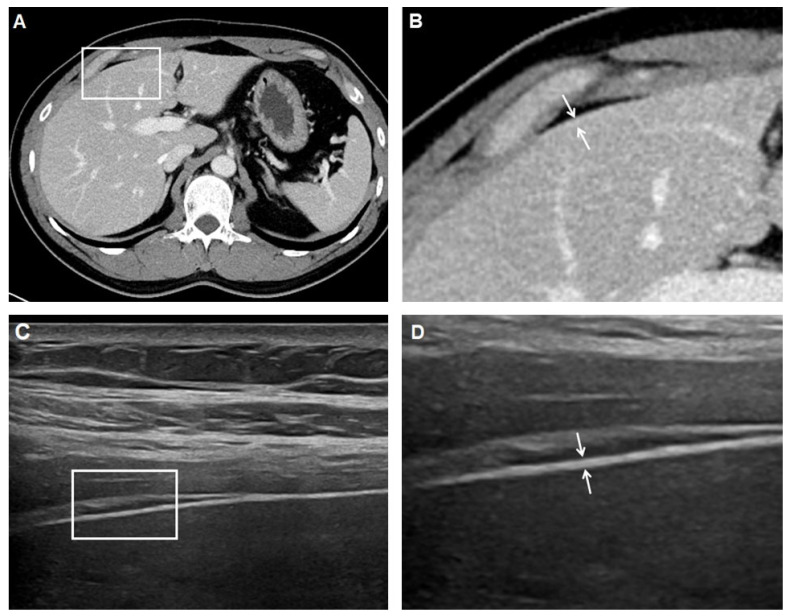
Measurement of hepatic capsular thickness on CT and US images. (**A**,**B**), Arterial-phase CT images. (**A**) Axial CT scan shows the region of interest (white box) at the level of the right and left portal vein bifurcation, which serves as the anatomical landmark for cross-modality correlation. (**B**) Magnified CT image demonstrates the measurement of the combined thickness of the hepatic capsule and visceral peritoneum between the arrows. (**C**,**D**), Corresponding US images. (**C**) Intercostal US scan identifies the anatomical location (white box) perpendicular to the anterior hepatic surface, correlated with the predefined CT landmarks. (**D**) Magnified US image shows the measurement of the capsular complex between the arrows.

For CT, measurements were standardized at the level of the bifurcation of the right and left portal veins, typically in segments V, VI, or VIII. For US, measurements were obtained perpendicular to the anterior hepatic surface on intercostal scans and were matched as closely as possible to the corresponding CT measurement site (Figure 1). For each modality, three repeated measurements were obtained by each reader, and the averaged values were used for analysis. When the hepatic capsule was not discretely visualized on CT, a baseline value of 0.5 mm was assigned for operational consistency in measurement, with reference to previously reported thickness data for the liver capsule and peritoneal layers [15,16,17]. This assumption should be interpreted cautiously because direct CT-based validation of this value is limited.

### 2.4. Statistical Analysis

All statistical analyses were performed using MedCalc software (version 17.1, MedCalc Software Ltd., Ostend, Belgium). HCT values were compared between the FHCS and control groups using the Mann–Whitney U test. The association between CT- and US-based HCT measurements was evaluated using Spearman correlation analysis. Interobserver agreement between the two readers for HCT measurement was assessed using the intraclass correlation coefficient (ICC), based on a two-way random-effects model with absolute agreement for single measurements. Receiver operating characteristic (ROC) curve analysis was performed to explore candidate cutoff values for discriminating FHCS from controls. A two-sided *p* value < 0.05 was considered statistically significant.

## 3. Results

Baseline clinical and imaging characteristics of the study population are summarized in Table 1. BMI and CRP were not included because these variables were unavailable or inconsistently documented, particularly in the control group. Leukocytosis was assessed in the FHCS group as part of the clinical diagnostic criteria, but comparable leukocyte data were not consistently available in the healthy control group.

### 3.1. Comparison of HCT Between Groups

**Figure 2:**
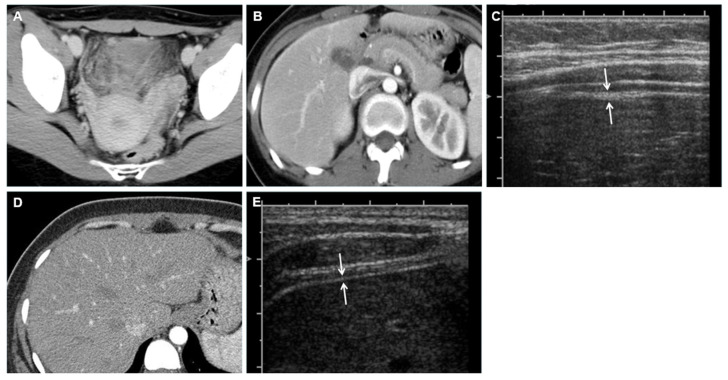
Representative CT and US images illustrating HCT in a patient with Fitz–Hugh–Curtis syndrome (FHCS) and a healthy control. (**A**–**C**), Images from an 18-year-old woman with FHCS. (**A**) Axial pelvic CT image demonstrates diffuse reticular infiltration within the pelvic intraperitoneal fat, consistent with pelvic inflammatory disease. (**B**) Contrast-enhanced axial CT image at the level of the liver shows thick enhancement along the anterior hepatic capsule. The combined thickness of the hepatic capsule and visceral peritoneum is measured between the arrows at the standardized anatomical landmark. (**C**) Corresponding abdominal US image obtained 1 day after CT using an 8 MHz linear transducer demonstrates measurement of the hepatic capsular complex (between the arrows) at maximum magnification. (**D**,**E**) images from a 25-year-old healthy woman evaluated as a kidney donor. (**D**) Axial CT image shows no discrete visualization of the hepatic capsule; therefore, a baseline thickness of 0.5 mm is assigned according to the predefined measurement protocol. (**E**) Corresponding abdominal US image demonstrates measurement of HCT (between the arrows) using the same standardized protocol as applied in the FHCS group.

**Figure 3:**
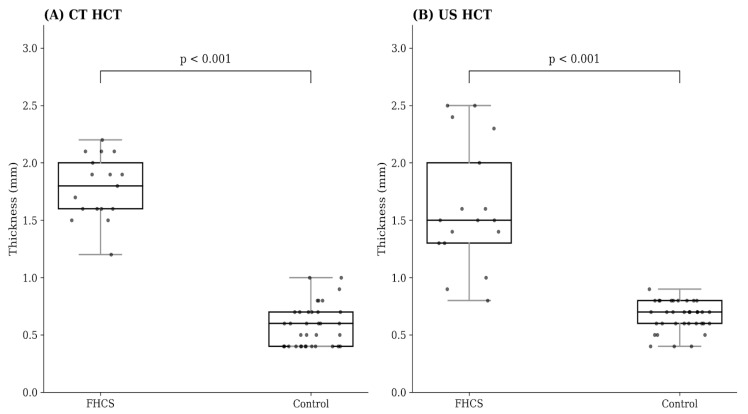
Comparison of HCT between patients with FHCS and controls on (**A**) CT and (**B**) US. Box plots represent the median and interquartile range. Individual data points are superimposed as jitters. *p* values were calculated using the Mann–Whitney U test. CT, computed tomography; FHCS, Fitz–Hugh–Curtis syndrome; HCT, hepatic capsular thickness; and US, ultrasonography.

HCT differed significantly between the FHCS group and the control group on both CT and US. On CT, median HCT was significantly greater in the FHCS group than in the control group [1.80 mm (IQR, 1.60–2.00) vs. 0.60 mm (IQR, 0.40–0.70); U = 595.0, *p* < 0.001]. On US, median HCT was also significantly greater in the FHCS group than in the control group [1.50 mm (IQR, 1.30–2.00) vs. 0.70 mm (IQR, 0.60–0.80); *U* = 589.0, *p* < 0.001] (Figure 2 and Figure 3; Table 1).

### 3.2. Correlation Between CT and US Measurements

**Figure 4:**
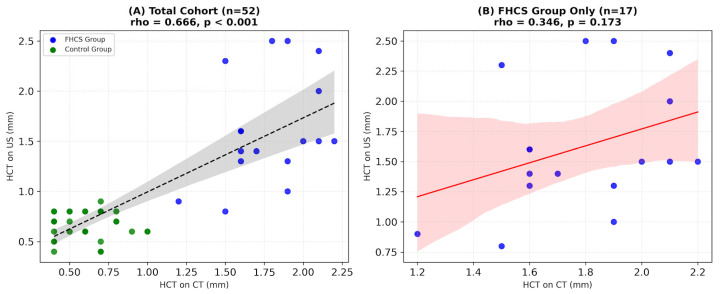
Spearman correlation analysis between CT- and US-based hepatic capsular thickness (HCT) measurements. (**A**) Overall cohort showing a significant positive correlation between CT and US measurements. (**B**) FHCS subgroup analysis showing a non-significant potential positive association. CT, computed tomography; FHCS, Fitz–Hugh–Curtis syndrome; HCT, hepatic capsular thickness; and US, ultrasonography.

CT- and US-based measurements of HCT showed a significant positive correlation across the overall cohort (rho = 0.666, *p* < 0.001) (Figure 4A). To address potential inflation of the correlation coefficient due to group separation, a subgroup analysis was performed. In the FHCS group, a potential positive association was observed (rho = 0.346, *p* = 0.173), but it did not reach statistical significance (Figure 4B). This lack of significance is likely attributable to the small sample size and restricted range of thickness measurements within the FHCS cohort. Therefore, while the cross-modality correlation was significant across the overall cohort, the correlation within the diseased population remains unproven in this sample and should be interpreted with caution.

### 3.3. Interobserver Agreement for HCT Measurement

Interobserver agreement for HCT measurement was good in the overall cohort, with an ICC of 0.804 (95% confidence interval [CI], 0.66–0.89). In subgroup analysis, the ICC was 0.535 (95% CI, 0.07–0.81) in the FHCS group, indicating moderate agreement, whereas the ICC was −0.087 (95% CI, −0.40–0.27) in the control group (Table 2).

### 3.4. Exploratory ROC Analysis and Candidate Cutoff Values

In exploratory ROC analysis, the area under the ROC curve (AUC) was 1.000 (95% CI: 1.000–1.000) for CT and 0.990 (95% CI: 0.964–1.000) for US. However, these values likely represent a theoretical maximum separation within an idealized case–control setting rather than real-world diagnostic performance. The candidate cutoff value for discriminating FHCS from controls was 1.1 mm for CT, corresponding to a sensitivity of 100% (95% CI: 80.5–100.0%) and a specificity of 100% (95% CI: 90.0–100.0%). For US, the candidate cutoff value was 0.85 mm, corresponding to a sensitivity of 94.1% (95% CI: 71.3–99.9%) and a specificity of 97.1% (95% CI: 85.1–99.9%) (Table 3).

## 4. Discussion

In this retrospective dual-center case–control study, we found that hepatic capsular thickness (HCT) measured on both arterial-phase CT and ultrasonography (US) was significantly greater in women with clinically diagnosed Fitz–Hugh–Curtis syndrome (FHCS) than in healthy controls. In addition, HCT measured on US showed a significant positive correlation of moderate strength with HCT measured on CT. These findings suggest that quantitative assessment of HCT on US may reflect perihepatic inflammatory change in FHCS and may provide supportive imaging information in selected clinical settings.

FHCS has traditionally been diagnosed on the basis of clinical presentation, microbiologic or serologic evidence of PID-related infection, and, in some cases, laparoscopic confirmation [18,19,20,21]. In recent decades, contrast-enhanced CT has emerged as a useful noninvasive imaging modality because capsular enhancement along the anterior hepatic surface can serve as a suggestive radiologic clue [1,2,3,4,22]. However, CT findings are not entirely specific, and similar perihepatic enhancement may also be encountered in other inflammatory or neoplastic peritoneal conditions [18,23,24,25]. Accordingly, imaging findings should always be interpreted in conjunction with the clinical and laboratory context.

Compared with CT, US has several practical advantages, including wide availability, low cost, and the absence of radiation exposure. However, unlike CT, US criteria for FHCS have not been clearly established. The prior sonographic literature has largely consisted of case reports or small descriptive series, and quantitative thresholds have not been well defined [5,10,11,12,13,14,26]. Our results extend the existing literature by suggesting that HCT can be measured on US and that increased US-based HCT is associated with FHCS in comparison with a control population.

The present findings should, however, be interpreted with caution. First, the study population was small, particularly within the FHCS cohort (*n* = 17), which limits the generalizability of our findings. Second, because the control group consisted of healthy women rather than patients with PID without FHCS or patients with other causes of right upper quadrant pain, the discriminative performance of HCT may have been overestimated. In routine clinical practice, the key diagnostic challenge is differentiating FHCS from other acute abdominal or pelvic inflammatory conditions rather than from healthy individuals. Therefore, the high AUC values and proposed cutoff thresholds should be regarded as exploratory and preliminary rather than definitive. This approach was adopted because of the retrospective study design and the requirement for both arterial-phase CT and abdominal US within a short interval, but it does not fully reflect the real-world diagnostic setting. Third, FHCS was diagnosed clinically and radiologically on the basis of compatible symptoms, leukocytosis, clinical response to antibiotic treatment, and characteristic arterial-phase CT findings, rather than according to a uniform microbiologic, serologic, or laparoscopic reference standard in all patients. Accordingly, our study should be interpreted as an evaluation of imaging association and discriminative performance within a clinically defined cohort, rather than as a definitive diagnostic accuracy study against a universal gold standard.

Several additional methodological limitations should also be acknowledged. HCT measurement on US is inherently operator-dependent and may be influenced by transducer selection, scanning angle, patient body habitus, and respiratory motion. In addition, CT-based measurement of such a thin structure is technically challenging. Although we attempted to standardize the measurement location, small errors in measurement may still have occurred. In the present study, interobserver agreement for HCT measurement was good in the overall cohort but was substantially lower in the control group. This finding may be explained by the very small and narrow range of thickness values in healthy controls, in which even minor absolute measurement differences between readers can markedly reduce the ICC. More importantly, the poor agreement in the control group highlights the inherent technical difficulty of measuring extremely thin, non-thickened capsular structures near the lower limit of current imaging resolution. Therefore, HCT should be interpreted cautiously as a quantitative imaging biomarker, particularly within the normal or near-normal range. Prospective investigations using predefined transducer selection and standardized measurement protocols are needed to improve reproducibility.

Despite these limitations, our study suggests that HCT measurement on US may have practical value as a supportive imaging marker in women with suspected FHCS. In particular, when CT is undesirable or not immediately available, a focally increased anterior hepatic capsular thickness on US, interpreted together with the clinical suspicion of PID-related perihepatitis, may increase diagnostic confidence. However, our data do not support replacing CT with US in all patients, and US findings should not be used in isolation.

## 5. Conclusions

HCT measured on US was significantly greater in women with clinically diagnosed FHCS than in healthy controls and showed a significant positive correlation of moderate strength with CT-based measurements. These results suggest that US-based HCT assessment may serve as a useful adjunctive imaging marker in suspected FHCS. However, given the small FHCS cohort and the use of healthy controls, the proposed cutoff values should be regarded as exploratory. Larger prospective studies including clinically relevant comparison groups are needed to validate the reproducibility, optimal threshold, and real-world diagnostic utility of this approach.

## Figures and Tables

**Table 1:** Baseline clinical and imaging characteristics of the study population.

	FHCS Group (*n* = 17)	Control Group (*n* = 35)	*p* Value
Age (years)	29.0 (22.0–32.0)	40.0 (35.5–45.0)	<0.001
CT-measured HCT (mm)	1.80 (1.60–2.00)	0.60 (0.40–0.70)	<0.001
US-measured HCT (mm)	1.50 (1.30–2.00)	0.70 (0.60–0.80)	<0.001

Data are presented as median (interquartile range), as appropriate. BMI and CRP were unavailable or inconsistently documented and were therefore not included. CT, computed tomography; FHCS, Fitz–Hugh–Curtis syndrome; HCT, hepatic capsular thickness; IQR, interquartile range; US, ultrasonography.

**Table 2:** Interobserver agreement (ICC) for HCT measurement.

Group	ICC (95% CI)	Agreement Level ^1^
Overall Cohort	0.804 (0.66–0.89)	Good
FHCS group	0.535 (0.07–0.81)	Moderate
Control Group	−0.087 (−0.40–0.27)	Poor (None)

^1^ Agreement levels based on Koo and Li’s guideline (Poor < 0.5, Moderate 0.5–0.75, Good 0.75–0.9, Excellent > 0.9).

**Table 3:** Exploratory ROC analysis of HCT on CT and US for discriminating Fitz–Hugh–Curtis syndrome from controls.

HCT	AUC	Cutoff (mm) ^1^	Sensitivity (%)	Specificity (%)
CT	1.000	0.85	100	91.4
		0.95	100	94.3
		1.1	100	100
		1.35	94	100
		1.45	94	100
US	0.990	0.65	100	42.9
		0.75	100	71.4
		0.85	94.1	97.1
		0.95	88.2	100
		1.15	82.4	100

^1^ The optimal cut-off values were determined using the Youden index to maximize the sum of sensitivity and specificity. Diagnostic performance is evaluated in 17 patients with FHCS and 35 controls. Data in the table represent values with 95% confidence intervals (CIs). AUC = area under the receiver operating characteristic curve; CT = computed tomography; HCT = hepatic capsular thickness; ROC = receiver operating characteristic; and US = ultrasonography.

## Data Availability

The data presented in this study are available upon request from the corresponding author. The data are not publicly available for confidentiality reasons.
